# Army Veterinary Bill

**Published:** 1894-03

**Authors:** 


### EDITORIAL.

### ARMY VETERINARY BILL.

The friends of the army veterinarians are solicited to use their efforts in obtaining the passage of the bill below, which has been introduced in the Senate and House of Representatives at Washington, as follows:

| | | |
|---|---|---|
| *Senate.* | Cameron, Penna | 991 |
| " | Pettigrew, S. D | 1489 |
| " | Kyle, S. D | 1456 |
| *House.* | Robinson, Penna | 4217 |
| " | Pickler, S. D | 5308 |

AN ACT TO FIX THE PAY, ALLOWANCES, PENSIONS, RETIREMENT AND RANK OF THE VETERINARIANS OF THE UNITED STATES ARMY.

*BE IT ENACTED by the Senate and House of Representatives of the United States of America, in Congress assembled:*

SECTION I. That the United States Army Veterinarian shall have the pay, allowances, pensions, retirements, tenure of office, and the relative rank of Second Lieutenant of Cavalry; their appointment to date from original entry into the Army. *Provided*, however, that hereafter, candidates for the United States Army Veterinary service shall comply with all the preliminaries now required from candidates for "The Army Medical Corps," and shall pass such examinations as the Secretary of War may direct.

SECTION II. This Act shall be in force and effect from, and after, its approval.

SECTION III. All Acts inconsistent with this Act are hereby repealed.

Each and every veterinarian and everyone interested in veterinary medicine will not only be doing a pleasant duty to the army veterinarians, but will be doing a great deal toward advancing the status of the veterinary profession, by aiding the passage of this bill. It is a disgrace that the United States alone of all governments fails to place the veterinarian on the list of commissioned officers. While they recognize him only on the level of a farrier, they can only expect to find enthusiasts who are willing to assume the positions, and they cannot expect the free, loyal service which they could from properly recognized and rewarded men.

Let each and every one write to his Senator and Representative, and secure his support and individual influence for the passage of this bill.

Send them petitions signed by every horseman in your locality, and request them to present them to the Military Committee of the Senate and House.

Tear out advertisement pages xiii and xv, to use as petitions and forward to your Representative and Senator.

